# Type 2 diabetes is associated with impaired jejunal enteroendocrine GLP-1 cell lineage in human obesity

**DOI:** 10.1038/s41366-020-00694-1

**Published:** 2020-10-09

**Authors:** Céline Osinski, Léa Le Gléau, Christine Poitou, Juan de Toro-Martin, Laurent Genser, Magali Fradet, Hédi Antoine Soula, Armelle Leturque, Corinne Blugeon, Laurent Jourdren, Edwige Ludiwyne Hubert, Karine Clément, Patricia Serradas, Agnès Ribeiro

**Affiliations:** 1 Sorbonne Université, INSERM, Nutrition and obesities: systemic approaches, F-75013 Paris, France; 2 Nutrition Department, Pitié-Salpêtrière hospital, Assistance Publique/Hôpitaux de Paris, F-75013, Paris, France; 3 Sorbonne Université, Université de Paris, INSERM, Cordeliers Research Center, F-75006 Paris, France; 4 Hepato-Biliary-Pancreatic Gastrointestinal Surgery and Liver Transplantation, Pitié-Salpêtrière Hospital, Assistance Publique/Hôpitaux de Paris, F-75013 Paris, France; 5 Cytometry platform, Institut Cardiometabolism and Nutrition, F-75013 Paris, France; 6 Genomics core facility, Département de biologie, Institut de Biologie de l’ENS (IBENS), École normale supérieure, CNRS, INSERM, Université PSL, 75005 Paris, France; 7 Present Address: Institute of Nutrition and Functional Foods (INAF), School of Nutrition, Université Laval, Quebec, QC Canada; 8 Present Address: Institut de Biologie, CIRB, Collège de France, F-75005 Paris, France; 9 Present Address: SERVIER, ADIR, F-92284 Suresnes, cedex France

**Keywords:** Obesity, Type 2 diabetes

## Abstract

**Objectives:**

Altered enteroendocrine cell (EEC) function in obesity and type 2 diabetes is not fully understood. Understanding the transcriptional program that controls EEC differentiation is important because some EEC types harbor significant therapeutic potential for type 2 diabetes.

**Methods:**

EEC isolation from jejunum of obese individuals with (ObD) or without (Ob) type 2 diabetes was obtained with a new method of cell sorting. EEC transcriptional profiles were established by RNA-sequencing in a first group of 14 Ob and 13 ObD individuals. EEC lineage and densities were studied in the jejunum of a second independent group of 37 Ob, 21 ObD and 22 non obese (NOb) individuals.

**Results:**

The RNA seq analysis revealed a distinctive transcriptomic signature and a decreased differentiation program in isolated EEC from ObD compared to Ob individuals. In the second independent group of ObD, Ob and NOb individuals a decreased GLP-1 cell lineage and GLP-1 maturation from proglucagon, were observed in ObD compared to Ob individuals. Furthermore, jejunal density of GLP-1-positive cells was significantly reduced in ObD compared to Ob individuals.

**Conclusions:**

These results highlight that the transcriptomic signature of EEC discriminate obese subjects according to their diabetic status. Furthermore, type 2 diabetes is associated with reduced GLP-1 cell differentiation and proglucagon maturation leading to low GLP-1-cell density in human obesity. These mechanisms could account for the decrease plasma GLP-1 observed in metabolic diseases.

## Introduction

Enterohormones, such as peptide YY (PYY), glucagon-like peptide-1 (GLP-1) cholecystokinin (CCK) and glucose-dependent insulinotropic peptide (GIP) control food intake and energy homeostasis in healthy individuals. How they influence the development of obesity is still debated. Obese individuals present an imbalance in these plasma entero-hormone [1]. Gastric bypass surgery is efficacious in reducing body weight and associated comorbidities while improving related metabolic parameters. The rise of post-prandial GLP-1 secretion after Roux-en-Y gastric bypass (RYGB) may improve metabolic parameters in type 2 diabetes (T2D) individuals [2–6]. One mechanism for improving the GLP-1 secretion after RYGB is due to the arrival of the nutrient flow in a more distal region, following reshaping of the intestine after surgery [7]. Therefore, the mechanisms driving the alteration of enterohormone secretion in obesity and after RYGB might not be restricted to GLP-1 incretin effects alone [4].

The gut epithelium adapts rapidly to environmental changes with complete cell renewal every 3–5 days in humans [8]. From progenitors located in crypts, cell differentiation is orchestrated by a network of transcription factors with specific contributions. It was reported that Notch signaling [9] and MATH1 expression [10] drive intestinal progenitors toward the secretory lineage. Then, NGN3 transcription factor restricts cells to the endocrine lineage [11, 12]. FOXA1/FOXA2 and NEUROD1 act downstream NGN3 to commit cells into either D (somatostatin)- and L (GLP-1)-cell phenotypes or into the I (CCK)- and S (secretin)-cell phenotypes, respectively [13]. Within enteroendocrine cells (EEC), some transcription factors, such as PAX4, PAX6 and NEUROD1, trigger enterohormone gene transcription [10]. Moreover, NEUROD1 [14] and ARX [15] have also been identified as in vivo regulators of EEC differentiation. The recent identification of EEC lineage regulators in real-time unveils a higher complexity in the transcription factor network [16]. Transcription factors involved in EEC differentiation have distinct temporal expression profiles allowing to be classified into early and common (NGN3, SOX4, PAX4, ARX…), middle and late (PAX6, FOXA1, TRIM35...) [16]. Recent data demonstrate that an EEC can co-express more than one hormone, rendering the alphabetical classification obsolete [17–19] and a new EEC classification was proposed [20]. However, most of these data were obtained in mice. A previous study in human duodenum showed a deregulation of transcriptional factors controlling intestinal cell differentiation in morbidly obese subjects [21]. Although a transcriptomic analysis of EEC in humans has been published recently [22], data in jejunum of subjects with severe obesity and T2D have not yet been reported.

Suspecting that nutritional changes can modulate EEC differentiation and contribute to the modulation of gut hormone secretion in metabolic diseases, we previously showed a positive association between GLP-1-positive cell density in jejunum samples and fat-consumption in individuals with severe obesity [23]. Beside nutrient sensing, enterohormone secretion is dependent on nutrient absorption [20]. Within small intestine, jejunum is one of the major absorption sites. Taking advantage of jejunal samples collected during bariatric surgery, we examined the impact of obesity and T2D on human EEC using an integrated strategy combining transcriptomic profiles, cell lineage gene expression and cell density analysis.

## Subjects and methods

The study was conducted in accordance with Helsinki Declaration, received approval from the local ethics committee (CPP Ile de France I) and was registered on the ClinicalTrials.gov website NCT01454232 and NCT02292121. Informed written consent was obtained from all individuals prior to study inclusion.

### Human individuals and jejunum sampling

This study is ancillary to previously published studies [24, 25] that included individuals with severe obesity involved in a bariatric surgery program carried out at the Pitié-Salpêtrière University Hospital, Nutrition and Visceral Surgery Departments (Paris, France). Medical history and clinical variables were recorded all patients before the gastric bypass surgery as described [25]. Patients were managed without any specific diet (ketogenic diet or other) or any change in the antidiabetic treatment just prior surgery. Patients have to be weight-stable before the intervention. Obese individuals were stratified according to their metabolic status, and two groups were constituted 1/ for EEC enrichment by FACS and transcriptomic, obese individuals without or with T2D (Ob, *n* = 14 and ObD, *n* = 13, Table 1) and 2/ for jejunal epithelial study by RT-qPCR, obese individuals without or with T2D (Ob, *n* = 37 and ObD, *n* = 21, Table 2). ObD individuals (fasting blood glucose >7 mmol/L and/or 11.1 mmol/L 2 h after a 75 g glucose load) received antidiabetic treatments or yet untreated. Antidiabetic treatments in ObD groups are metformin and/or sulfonylurea and/or GLP-1 agonists or DDP4-inhibitors and/or insulin as described in Tables 1 and 2.

**Table 1:** Clinical and biological baseline characteristics of non-obese (NOb), obese (Ob) and obese-diabetic (ObD) individuals.

	Individuals	NOb	Ob	ObD	*p* value		
Demographic data	*n*	22	37	21	*Ob/NOb*	*ObD/NOb*	*ObD/Ob*
	Age (years)	61.7 ± 2.01	41.2 ± 1.4	50.4 ± 1.5	8 e-10	0.014	0.002
	Sex (F/M)	7/15	31/6	11/10	0.0007		
Corpulence	BMI (Kg/m^2^)	22.7 ± 0.5	45.8 ± 0.9	45.6 ± 1.3	<0.0001 (NOb vs. Ob/ObD)		
	Lean mass (%)	nd	48.4 ± 0.6	43.4 ± 0.9			0.0022
	Fat mass (%)	nd	49.3 ± 0.6	54.1 ± 0.9			0.0021
Glucose metabolism	Glucose (mmol/L)	6.13 ± 0.26	4.99 ± 0.07	8.47 ± 0.50	0.089	5.3 e-04	3.7 e-9
	Insulin (µU/mL)	nd	18.76 ± 1.39	nd			
	HbA_1c_ (%)	nd	5.64 ± 0.06	8.20 ± 0.24			0.0001
	HOMA-IR	nd	4.2 ± 0.7	nd			
Lipid metabolism	Triglyceride (mmol/L)	nd	1.37 ± 0.10	2.63 ± 0.21			0.0004
	Cholesterol (mmol/L)	nd	5.19 ± 0.14	4.49 ± 0.17			0.0334
	HDL (mmol/L)	nd	1.17 ± 0.10	0.94 ± 0.05			0.0168
	LDL (mmol/L)	nd	3.23 ± 0.23	2.48 ± 0.18			0.0071
Comorbidities	T2D diabetes (*n*)	0	0	21			
	T2D treatment (%)	0	0	90.5			
	T2D treatment (*n*b)			2 (0–4)			
	Without treatment (*n*)			2			
	Mono treatment (*n*)	0	0	5			
	Combination of treatments (*n*)			14			
	T2D duration (year)			8.74 ± 1.6 (0–35)			
	Dyslipidemia treatment (%)	0	8.1	57.1			
	Hypertension (%)	22.7	27.0	80.9			

ObD patients (fasting blood glucose >7 mmol/L and/or 11.1 mmol/L 2 h after a 75 g glucose load) were treated with metformin and/or sulfonylureas and/or GLP-1 agonists and/or insulin. ObD individuals (*n* = 21) were untreated (*n* = 2), treated with monotherapy (*n* = 5), or combination therapy (*n* = 14). Among them, 52% individuals were taking DPP4-inhibitors or GLP-1 receptor agonists (*n* = 11) and 43% insulin therapy (*n* = 4). Mean ± SEM (min–max).

*T2D* type 2 diabetes.

**Table 2:** Clinical and biological baseline characteristics of obese (Ob) and obese-diabetic (ObD) individuals.

	Individuals	Ob	ObD	*p* value
Demographic data	*n*	14	13	
	Age (years)	45.36 ± 2.17	49.69 ± 3.22	0.38
	Sex (F/M)	13/1	6/7	0.0056
Corpulence	Weight (Kg)	127.62 ± 3.76	125.05 ± 4.3	0.833
	BMI (Kg/m^2^)	46.47 ± 1.02	43.90 ± 1.53	0.23
	Lean mass (%)	46.45 ± 0.85	52.19 ± 1.42	0.0098
	Fat mass (%)	51.32 ± 0.87	45.41 ± 1.45	0.0098
Glucose metabolism	Glucose (mmol/L)	5.04 ± 0.12	8.18 ± 0.62	0.0011
	Insulin (µU/mL)	18.90 ± 2.53	26.20 ± 1.76^a^	0.2532
	HbA_1c_ (%)	5.64 ± 0.09	7.43 ± 0.24	1.27e-05
Lipid metabolism	Triglyceride (mmol/L)	1.48 ± 0.13	1.77 ± 0.19	0.3294
	Cholesterol (mmol/L)	4.97 ± 0.11	4.29 ± 0.17	0.0119
	Glycerol (mmol/L)	0.11 ± 0.01	0.086 ± 0.007	0.360
	Free fatty Acids (mmol/L)	0.53 ± 0.05	0.066 ± 0.07	0.283
	HDL (mmol/L)	1.21 ± 0.05	1.04 ± 0.04	0.0536
	Apo A-I (g/L)	1.45 ± 0.04	1.38 ± 0.04	0.298
	LDL (mmol/L)	3.09 ± 0.12	2.45 ± 0.17	0.0180
	Apo B (g/L)	0.94 ± 0.04	0.88 ± 0.05	0.267
Low-grade inflammation	CRP (mg/L)	7.21 ± 1.26	7.395 ± 1.36	0.940
	Orosomucoïd (mg/L)	0.92 ± 0.05	0.89 ± 0.07	0.817
	Haptoglobin (mg/L)	1.69 ± 0.172	1.65 ± 0.109	0.863
	IL-6 (pg/mL)	3.87 ± 0.31	7.24 ± 1.11	0.173
Adipokines	Leptin (ng/mL)	73.34 ± 7.89	62.31 ± 6.89	0.432
	Adiponectin (µg/mL)	4.98 ± 0.48	3.58 ± 0.298	0.086
Comorbidities	T2D treatment (%)	0	76.9	
	T2D treatments (*n*b)		1 (0–4)	
	Without treatment (*n*)		3	
	Mono treatment (*n*)	0	5	
	Combination of treatments (*n*)		5	
	T2D duration (years)		6.5 ± 1.49 (0–20)	
	Dyslipidemia (%)	78.6	84.6	
	Hypertension (%)	35.7	76.9	

ObD individuals (fasting blood glucose >7 mmol/L and/or 11.1 mmol/L 2 h after a 75 g glucose load) were treated with oral antidiabetic drugs and/or insulin and/or GLP-1 agonists.

ObD individuals (*n* = 13) were untreated (*n* = 3), treated with monotherapy (*n* = 5), or combination therapy (*n* = 5). Among them, 23% individuals were taking DPP4-inhibitors or GLP-1 receptor agonists (*n* = 3) and 31% on insulin therapy (*n* = 4).

*T2D* type 2 diabetes.

^a^Blood insulin levels in individuals not treated with exogenous insulin (*n* = 9). Mean ± SEM (min–max).

The use of a single group for all analyses was incompatible with the size of jejunum samples collected during bariatric surgery (4 cm). Indeed, EEC enrichment by FACS needs a large quantity of epithelial cells that does not allow to keep enough cells to perform RT-qPCR.

A group of non-obese individuals (NOb *n* = 22; body mass index (BMI) = 22.7 kg/m^2^ [range 17–27]) was constituted for jejunal epithelial study. Jejunal tissue from NOb individuals were jejunum biopsy donors (*n* = 3) (double balloon endoscopy; normal histopathology, absence of anti-inflammatory treatment) or jejunum samples taken at distance of pancreatic or gastric tumor during duodenal resection in absence of chemotherapy or apparent metabolic consequence (*n* = 19) (Table 2). Jejunum samples of NOb subjects with pancreatic or gastric tumor were collected at the same site as was performed RYGB in obese subjects, i.e., 60–70 cm distal to the ligament of Treitz as previously described [26]. We previously compared jejunum samples of Ob subjects to NOb [25, 27]. Importantly, NOb individuals with, renal- cardiac- or hepatic failure were not included in this study.

### Jejunal epithelial cells isolation

Proximal jejunal samples were collected during gastric bypass surgery and epithelial cells were prepared as previously described [27].

### FACS for enriched enteroendocrine cell preparation

For cell sorting experiments, 100.10^6^ intestinal epithelial cells were rinsed in FACS buffer (PBS, 3% FCS, 2 mM EDTA), blocked with Human Fc Receptor Binding Inhibitor Antibody (eBioscience) and stained with CD45-BV421 (Bio Legend) and CD24-PeCy7 (Bio Legend) antibodies. Dead cells were excluded with propidium iodure (eBiosciences). During sorting experiments, cells were placed in FACS buffer with RNAse inhibitor (Life Technologies). CD24 marker was used to select EEC, and CD45 was used to get rid of immune cells, especially B cells that also expressed CD24. CD45-negative, CD24-positive EEC were sorted on a jet-in-air flow cytometer (MoFlo Astrios, Summit software, Beckman Coulter). After FACS, an EEC enriched population was obtained containing 1.3.10^6^ cells.

The validation of the EEC sorting was performed in a limited number of independent subjects (*n* = 7). Gene expression in CD24+ cells was analyzed by RT-qPCR in 3 Ob subjects and protein expression was studied by Western Simple assay on 2 Ob and 2 ObD subjects.

### Protein analysis

Cell-sorted populations were lysed in ice-cold buffer (0.4% Triton 100X, 2 mM DTT, 5 µg/mL leupeptine, 0.4 mM PMSF). Protein homogenates (0.1 mg/mL) were analyzed by Simple Western assay (Wes^™^ instrument by ProteinSimple) for chromogranin A (Abcam) and actin (Novus).

### cDNA libraries and RNA sequencing

Total RNA from sorted-cells was extracted with RNAeasy Mini Plus Kit (Qiagen). RNA concentration and quality were assessed (2100 Bioanalyzer, Agilent Technologies). RIN values ranged between 6.0 and 8.2.

10 ng of total RNA were converted to cDNA using SMART-Seq v4 Ultra Low Input RNA kit (Clontech). An average of 150 pg of amplified cDNA was used to prepare libraries following instructions from the Nextera XT DNA kit (Illumina). Libraries were multiplexed by 28 on 2 high-output flow cells and sequenced on a NextSeq500 device (Illumina). A mean of 37 ± 8 million 75 bp reads passing Illumina quality filter reads were obtained for each of the 28 samples.

The analyses were performed using the Eoulsan pipeline [28]. Before mapping, poly N read tails were trimmed, reads ≤40 bases were removed, and reads with quality mean ≤30 were discarded. Reads were then aligned against the human genome from Ensembl version 88 using STAR (version 2.5.2b) [29]. Alignments from reads matching more than once on the reference genome were removed using Java version of samtools [30]. To compute gene expression, human GTF genome annotation version 88 from Ensembl database was used. All overlapping regions between alignments and referenced exons were counted using HTSeq-count 0.5.3 [31]. The RNA-Seq gene expression data and raw fastq files are available on the GEO repository (www.ncbi.nlm.nih.gov/geo/) under accession number GSE132831.

### Taqman low density arrays and RT-qPCR

Total RNA of epithelial cells was extracted with RNAeasy Mini Plus Kit (Qiagen). RNA concentration and quality were assessed (2100 Bioanalyzer, Agilent Technologies). The RIN value was 7–9.5. Total RNA (1 μg) was used to generate DNA with RT High Capacity (Thermofisher). cDNA (500 ng) was used for low-density-array (Applied Biosystem). 18 S gene was used for normalization. Quantitative real-time PCR was performed with SYBR Green PCR kits (Applied Biosystems) using Mx300p Stratagen system (Applied Biosystem). The cyclophilin gene was used for normalization. Relative quantification was determined using the 2^−ΔΔCt^ method.

### Immunohistochemistry for enteroendocrine cell density

Pieces of jejunum were fixed in formalin-acetic acid-alcohol before embedding in paraffin. For immunostaining (5 μm paraffin sections) primary antibodies were incubated for 1 h at room temperature (active GLP-1 7–36 amide (Peninsula), GIP (Abcam), CCK (Abcam), PYY (Peninsula)). Streptavidin Biotin Peroxidase kit (BioSpa) and DAB staining (DAKO) were used for revelation. Tissue sections were counterstained with hematoxylin. Images from all the slices (*n* = 187) corresponding to overall 2073 mm² of jejunal mucosae, were acquired using a Leica DMRB microscope coupled to a Leica DFC295 camera (software Leica Qwin 500). The number of GLP-1-, GIP-, CCK- and PYY-positive cells was quantified (ImageJ 1.46) per mm² mucosae (cell densities).

### Statistical analysis

Data are expressed as mean ± standard error of the mean (SEM) unless otherwise indicated. We used R software for all statistical analysis. For all comparisons, we used a generalized linear model (GLM) with Gaussian family classification, adjusting for age, sex and BMI (age and sex only when comparing obese and non-obese individuals). The relevant *p* values were obtained using ANOVA on the full models. GLM were computed using glmer and ANOVA functions in lme4 and car R package. To assess differential expression on semi-quantitative techniques such as RNAseq data, EdgeR (Biocomputing) package was used using negative binomial test. P values were adjusted using the false discovery rate (FDR) method with a threshold of 0.001. Only genes with count per million (cpm) over 100 for at least two individuals were considered. *p* < 0.05 was considered as statistically significance.

## Results

### Preparation of EEC populations from obese and obese-diabetic individuals

EEC being rare and scattered along the crypt-villus axis, we had to enrich the epithelial cell preparation in EEC using appropriate extracellular markers. Starting from total epithelial cells, CD45 marker was used to eliminate immune cells especially B cells which express CD24 marker. CD24 previously described in [32] was used to sort mostly EEC with also Paneth cells and stem cells (Supplementary Fig. 1a). This strategy of cell enrichment developed for human jejunum gives access to EEC with intact RNA (Supplementary Fig. 1b). To validate this new method of cell sorting, studies on CD45- CD24+ cells of seven independent subjects were carried out. A 50-fold EEC enrichment was obtained by measuring gene expression of specific markers of EEC, namely chromogranin A (CHGA) and proglucagon (GCG) in 3 Ob subjects (Supplementary Fig. 1c). By Simple Western assay, we confirmed the EEC enrichment showing the specific presence of CHGA in the CD24+ fraction, and its absence in the CD24- fraction in 4 subjects, 2 with T2D and 2 without (Supplementary Fig. 1d).

### Altered jejunal EEC transcriptomic profiles in obese individuals are associated with T2D

**Fig. 1:**
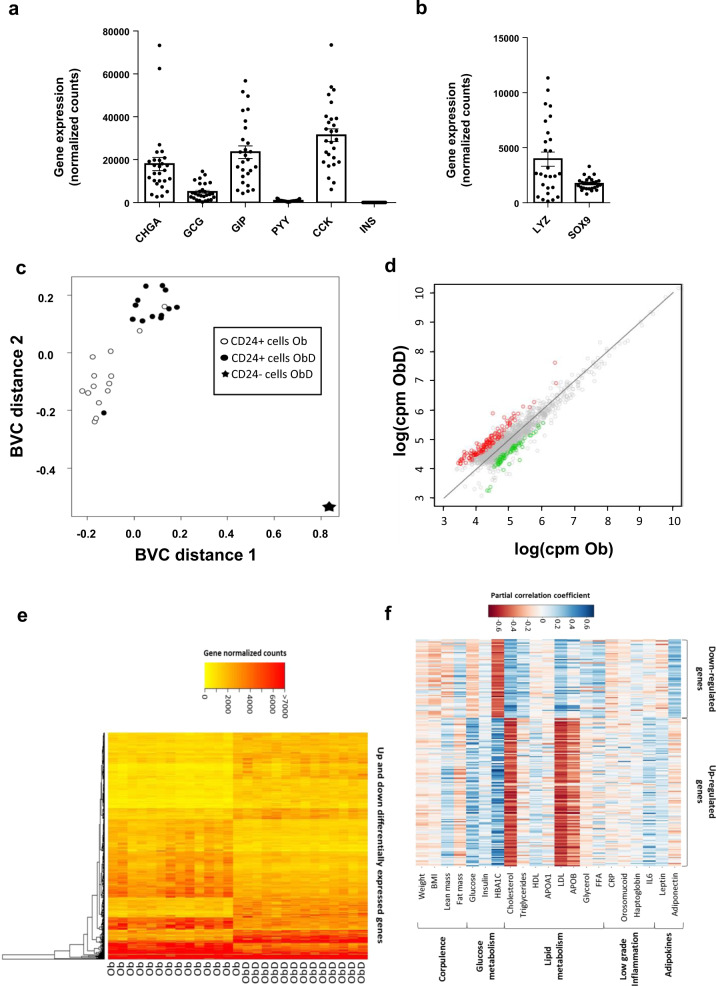
RNA sequencing analysis of human EEC from ObD and Ob individuals. RNA-seq analysis of gene expression of (**a**) EEC markers and (**b**) Paneth cell markers is expressed in normalized counts. Note that INS gene expression is a negative control of non expressed gene in EEC. **c** Multi-Dimensional Scaling of all differentially expressed genes in enriched-EEC population of Ob (*n* = 14, open circle) and ObD (*n* = 13, black circle) individuals and in ObD CD24 negative epithelial sample (*n* = 1, black star). **d** Comparative expression (counts per million) of genes present in enriched-EEC population between Ob and ObD individuals. Up-regulated (red) and downregulated (green) genes (FDR < 0.001, *n* = 196) are above and below the diagonal, respectively. **e** Hierarchical clustering from all the over-expressed (128 genes) and under-expressed (68 genes) genes (FDR < 0.001) in ObD compared to Ob individuals displayed as a heat map constructed on log (cpm). **f** Heat map of partial correlation (adjusted for age and sex) between clinical parameters and the differentially expressed genes (FDR < 0.001, *n* = 196).

EEC enrichment from 14 severely obese individuals without T2D (Ob) and 13 with T2D (ObD) (Table 1) was performed as previously described. RNAseq profiles of enriched EEC were determined (accession number GSE132831). For a single subject, the expression of genes of non-enteroendocrine CD24- and enteroendocrine CD24+ cells was analyzed (Supplementary Fig. 2). We showed that in CD24+ cells, the expression of enteroendocrine markers, CHGA, GCG, GIP CCK and PYY are overexpressed by 24-, 37-, 17-, 262- and 89-fold change, respectively. As expected, EEC and Paneth cell markers were expressed (Fig. 1a, b). The multidimensional scaling analysis showed that CD24+ EEC transcriptomic profiles are fully separated from that of CD24- cells (Fig. 1c). Furthermore, CD24+ EEC transcriptomic profiles from Ob subjects are well separated from ObD subjects (Fig. 1c). Differentially expressed genes in EEC from ObD vs. Ob individuals were shown in Fig. 1d, in which 128 upregulated genes and 68 downregulated genes with a FDR < 0.001 were indicated. The hierarchical clustering of these 196 differentially expressed genes further highlighted a clear distinction between ObD and Ob samples (Fig. 1e). Furthermore, partial correlation analyses showed that clinical parameters discriminate the down- and up-regulated genes in EEC (Fig. 1f). Thus, up- and downregulated genes, which are positively correlated with the biological parameters of diabetes (HbA1c, glycemia) are negatively correlated with the dyslipidemia parameters (cholesterol, LDL and apo B), and vice versa.

Altogether, the RNAseq analysis showed that transcriptomic signature of EEC discriminates obese subjects according to their diabetic status.

### Decreased EEC differentiation program in the jejunum of obese individuals is associated with T2D

**Fig. 2:**
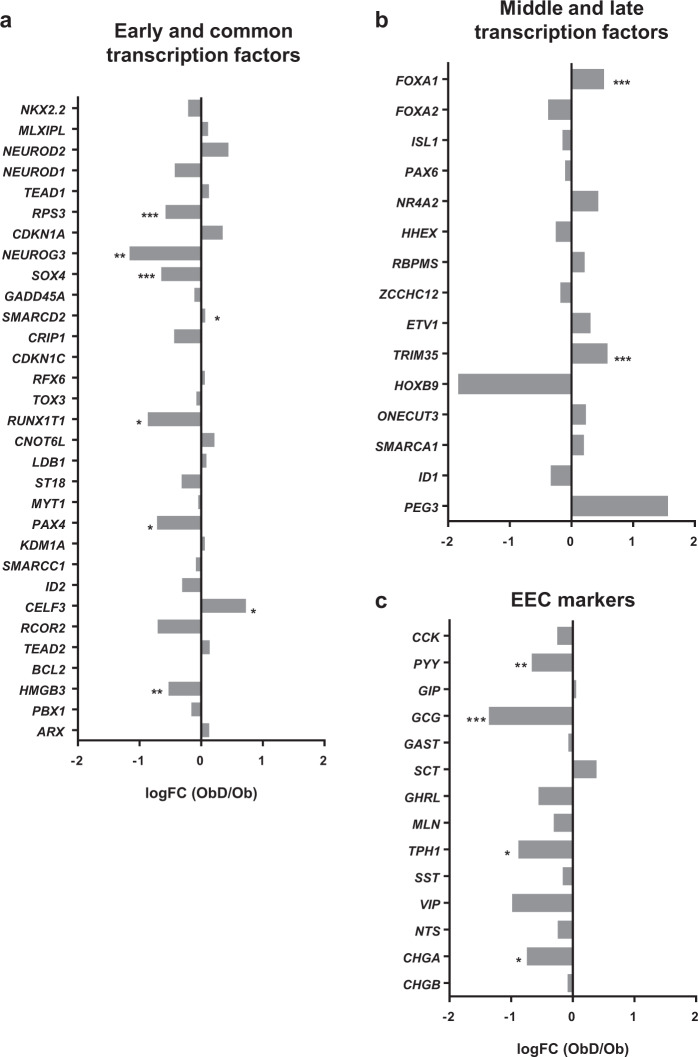
RNA-seq analysis of EEC lineage gene expression from ObD and Ob individuals. Gene expression of (**a**) early and common transcription factors, (**b**) middle and late transcription factors and (**c**) enteroendocrine cell markers. The fold change is calculated as the ratio of normalized counts ObD/Ob means. The gene expression is calculated as log(FC) from ObD (*n* = 13) and Ob (*n* = 14) individuals. *p* values were calculated with one way Anova. *, *p* < 0.05; **, *p* < 0.01; ***, *p* < 0.001.

Among early and common transcription factors, RPS3, NEUROG3, SOX4, RUN1XT1, PAX4 and HMGB3 were statistically decreased in ObD as compared to Ob individuals whereas SMARCD2 and CELF3 were increased (Fig. 2a). Regarding the 16 middle and late transcription factors, only FOXA1 and TRIM35 were increased in ObD as compared to Ob individuals (Fig. 2b). EEC markers were also analyzed in transcriptomic profiles. Gene expression of CHGA, PYY, GCG and tryptophan 5 hydroxylase 1 (TPH1), the serotonin production enzyme, were decreased in ObD compared to Ob (Fig. 2c). These highlighted the decreased EEC differentiation program associated with the diabetic status in human obesity.

### Type 2 diabetes is associated with a reduction of GLP-1 cell lineage in human obesity

**Fig. 3:**
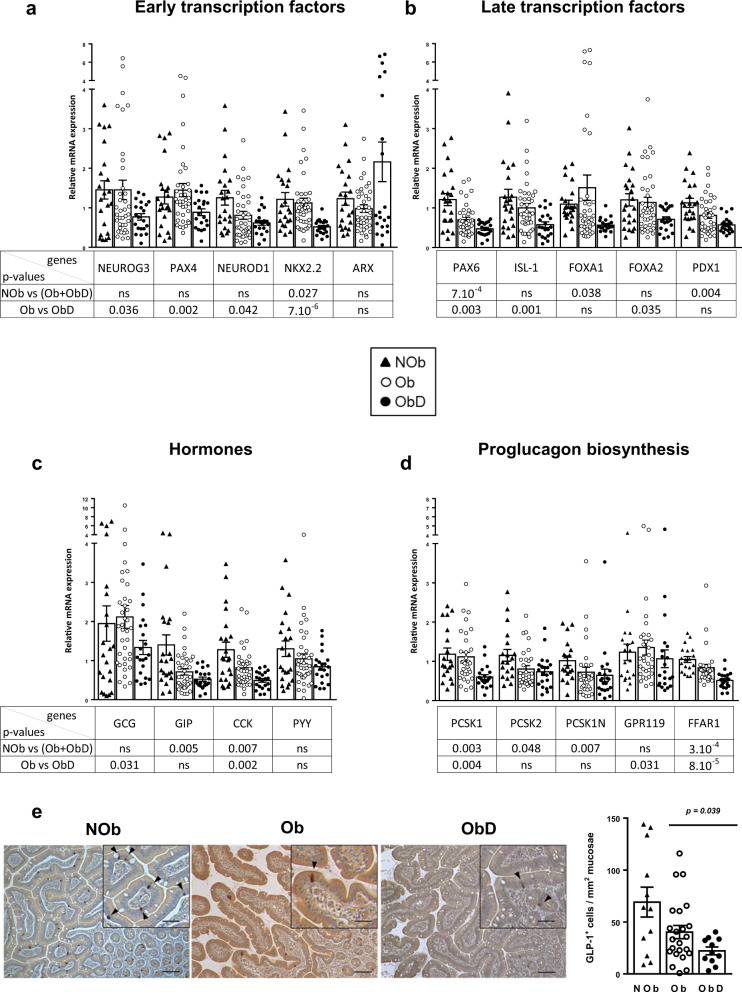
Impact of obesity and type 2 diabetes on GLP-1 cell lineage, GLP-1 biosynthesis and cell density. Gene expression analysis with Low Density Arrays of (**a**) early transcription factors, (**b**) late transcription factors involved in EEC differentiation, (**c**) enterohormones in NOb (*n* = 22), Ob (*n* = 37) and ObD (*n* = 21). **d** RT-qPCR analysis of gene expression involved in proglucagon maturation and lipid sensing triggering GLP-1 secretion in NOb (*n* = 19), Ob (*n* = 31) and ObD (*n* = 21). **e** Representative images of GLP-1 immunostaining in jejunum sections in which positive cells are colored in brown (scale bar 150 µm). A magnification is shown in the box (scale bar 300 µm) in which positive cells are indicated with black arrowheads. Note that an antibody against active GLP-1 7-36 amide form was used for the GLP-1 immunostaining. GLP-1 cell density is expressed as number of positive cells per mm^2^ of the jejunum mucosae in NOb (*n* = 12, black triangle), Ob (*n* = 23, open circle) and ObD (*n* = 11, black circle) individuals. Data are expressed as mean ± standard error of the mean (SEM).

**Fig. 4:**
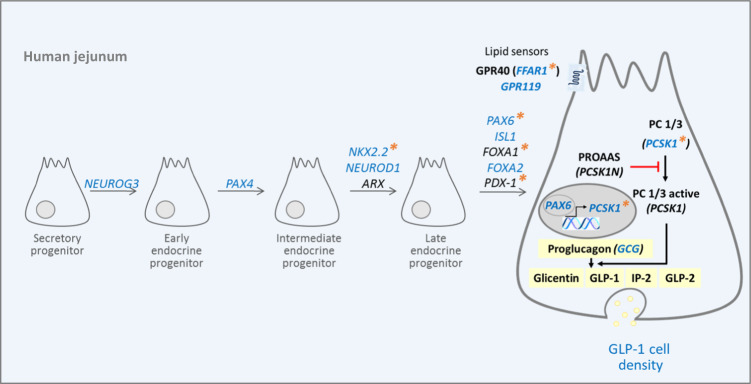
Diabetes in severe obese individuals specifically reduces the lineage and the density of jejunal GLP-1 producing cells. T2D in human obesity reduces both proglucagon *GCG* gene expression and GLP-1 cell density that could be related to a decrease in GLP-1 cell lineage factors (*NEUROG3*, *PAX4*, *NKX2.2*, *NEUROD1*, *PAX6*, *ISL1*, and *FOXA2)*. Furthermore, the expression of PCSK1, (encoding enzyme cleaving proglucagon into GLP-1) and lipid sensors, *GPR119* and *FFAR1* (leading to GLP-1 secretion) was also lowered by T2D. Blue color indicates decreased gene expression and lower GLP-1 cell density in ObD compared to Ob individuals; orange asterisks indicate decreased gene expression in obese (Ob + ObD) compared to NOb individuals.

We took advantage of a larger group of three groups of non obese (NOb, *n* = 22), Ob (*n* = 37), and ObD (*n* = 21) individuals (Table 2) to focus on the epithelial gene expression of ten transcription factors critical for the differentiation of EEC (Fig. 3a, b) and 4 enterohormones (Fig. 3c). We showed a significant lower expression of *NKX2.2*, *PAX6*, *FOXA1* and *PDX1* in obese without or with T2D (Ob + ObD) vs. NOb individuals (Fig. 3a, b). *GIP* and *CCK* gene expression were also decreased in obese without or with T2D (Ob + ObD) as compared to NOb individuals (Fig. 3c). When comparing ObD to Ob individuals, we found a significant decreased gene expression of *NEUROG3*, *PAX4*, *NEUROD1*, *NKX2.2*, *PAX6*, *ISL1* and *FOXA2* (Fig. 3a, b). *GCG* and *CCK* gene expression were also decreased in ObD compared to Ob (Fig. 3c). Altogether we showed in a second group, a specific reduction of CCK and GLP-1 cell lineages in obesity and T2D. Interestingly, obesity with T2D decreased GCG expression and its transcriptional regulator PAX6.

### Reduced proglucagon processing and lipid sensors involved in GLP-1 secretion in the epithelial jejunum of individuals with obesity and diabetes

To go further into mechanisms, we then focused on a panel of genes regulated by PAX6 and explored proglucagon maturation and sensors mediating lipid-induced GLP-1 secretion (Fig. 3d).

We observed a significant reduction of *PCSK1*, *PCSK1N*, and *PCSK2* expression in obese individuals without or with T2D (Ob + ObD) as compared to NOb (Fig. 3d). *PCSK1* gene expression was also significantly reduced in ObD individuals compared to Ob, thus suggesting a role of the metabolic status in proglucagon maturation (Fig. 3d). Surprisingly, the expression of *PCSK2*, involved in proglucagon maturation into glucagon in pancreatic alpha cells was detected in the jejunum at a level comparable to *PCSK1* (Ct 29.95 and Ct 29.31 for *PCSK2* and *PCSK1*, respectively).

Interestingly we analyzed gene expression of lipid sensors that induce GLP-1 secretion, namely *FFAR1* and *GPR119*. We observed a significant reduction of *FFAR1* gene expression in obese individuals without or with T2D (Ob + ObD) as compared to NOb. *FFAR1* and *GPR119* gene expression were both decreased in ObD as compared to Ob individuals (Fig. 3d).

Altogether, we showed that multiple aspects of GLP-1 synthesis, maturation and lipid sensing are negatively impacted by T2D in individuals with severe obesity.

### Decreased GLP-1-cell density in individuals with obesity and diabetes

We thus investigated if the alteration of GLP-1-cell density could be associated with diminished cell differentiation. GLP-1-, GIP-, CCK-, PYY-cell densities, according to enterohormone protein expression, were determined in NOb (*n* = 12), Ob (*n* = 23) and ObD (*n* = 11) individuals which came from the group of 58 individuals described in Table 2. GLP-1-, CCK-, GIP- and PYY-positive cells were identified by immunochemistry and their respective densities evaluated (Fig. 3e, Supplementary Fig. 3). Only the GLP-1-positive cell density was significantly decreased in ObD compared to Ob individuals (Fig. 3e). Thus, in the jejunum, diabetes specifically affects the density of GLP-1-positive EEC in individuals with severe obesity.

Altogether, we showed that obesity partly modifies the GLP-1 cell lineage in human jejunum whereas T2D deeply alters this lineage until the GLP-1 cell density is affected.

## Discussion

Using a new strategy of EEC sorting from human jejunum we provide for the first-time transcriptomic profiles of EEC in metabolic diseases. We showed that EEC transcriptomic signature discriminates obese individuals according to their diabetic status. A focus on EEC lineage showed a decrease in differentiation program in obesity and T2D. Our finding was further confirmed in a second group by a specific reduction of GLP-1-cell lineage and hormone synthesis. Furthermore, T2D in human obesity is associated with lowered GLP-1-positive cell density. Our findings are summarized in Fig. 4.

EEC are rare, scattered in the intestinal epithelium and difficult to isolate. In mice, EEC sorting is performed using enterohormone promoters driving a fluorescent tag. FACS has been used successfully for RNA sequencing experiments in transgenic mice [33, 34], but is not applicable in humans. Another strategy to isolate EEC is the use of EEC markers. While the surface marker Claudin 4 has been used once in mice [35] an intracellular cell marker, such as GLP-1 requires cell fixation and permeabilization prior sorting that might imply several drawbacks on cell viability and RNA integrity [22]. Here, in humans, CD24 was successfully used to enrich EEC. CD24 was previously used in mice to separate stem cells that weakly express CD24 from Paneth cells and EEC that strongly express CD24 [32]. Importantly, proteins and RNAs from human EEC-enriched fraction were of high quality allowing further exploration. Although we cannot exclude having selected a certain type of EEC, the RNA-seq analysis shows that no less than 12 enterohormones are present. Moreover, our technical stra-tegy preserved EEC differences according to the metabolic status of the individuals as we could distinguish between Ob and ObD EEC profiles.

Here, the expression of several genes involved in the EEC differentiation program is impacted in individuals with T2D. The expression of early transcription factors *(NEUROG3*, *PAX4*, *SOX4*, *RUNX1T1)* are lowered whereas late transcription factors (*FOXA1* and *TRIM35)* are increased in T2D individuals. Genetically modified mice lacking transcription factor expression have provided considerable knowledge on EEC lineage. NKX2.2 is a critical transcription factor involved in EEC lineage [36]. In addition, ARX, FOXA1, FOXA2, ISL-1 and PAX6 are important to promote GLP-1 lineage [13, 37–40]. High-fat diet (HFD)-induced obesity in mice reduces gene expression of *Etv1*, *Isl1*, *Mlxipl*, *Nkx2.2* and *Rfx6* whereas *Cck*, *Gip* and *Pyy*, *Gcg* gene expression is not impacted [41]. Wölnerhanssen et al. showed a deregulation of HES1, ATOH1, NEUROD1 and NEUROG3 controlling intestinal epithelial cell lineages in duodenum of obese individuals [21]. Recently, six new regulator genes were identified for EEC lineage in mice: *Sox4*, *Rfx6*, *Tox3*, *Myt1*, *Runx1t1*, and *Zcchc12* [16]. In our RNA-seq analysis, we also show that these genes are expressed in the EEC fraction but only SOX4 and RUNX1T1 expression is decreased in T2D individuals. Gehart et al. further showed that *Etv1*, *Hoxb9*, *Pax6* and *Trim35* genes are involved in GLP-1-cell lineage in mice [16]. Here, we show an increase of *TRIM35* in T2D individuals. In our study, we provide transcriptomic profiles of jejunal EEC in metabolic diseases, and we show that the EEC differentiation program is decreased.

Taken advantage of a second independent group of Ob and ObD individuals, including NOb individuals, we focused on the GLP-1 cell lineage. Obesity decreases the GLP-1 cell lineage as evidenced by lower *NKX2.2*, *PAX6*, *FOXA1*, and *PDX1* gene expression in (Ob + ObD) individuals compared to NOb individuals (Fig. 4). Moreover, GLP-1 cell lineage is altered by T2D status since *NEURO-G3*, *PAX4*, *NEUROD1*, *ISl-1* and *FOXA2* gene expression is decreased in ObD individuals compared to ObD individuals (Fig. 4).

We showed a decrease in gene expression of NEUROG3, PAX4 and GCG in the two independent groups of individuals. For PAX6 and PYY gene expression, we did not observe the same variation between the two groups. This could be explained by the smaller group 1 than group 2. Moreover, gene expression was not analyzed with the same samples (isolated EECs vs. total epithelial cells) nor the same methods (RNA-Sequencing vs. RT-qPCR).

We also report here that jejunal *GCG* gene expression is altered in T2D individuals that could indicate that the GLP-1-positive cells displayed lower capacity of GLP-1 biosynthesis. Indeed, the expression of *Pcsk1*, its inhibitor *Pcsk1n* and *Pcsk2* are under the control of PAX6 in pancreatic islets [42–44]. The *PCSK1* gene encodes prohormone convertase 1/3 (PC1/3), a protease involved in the post-translational processing of several prohormones in endocrine tissues. PC1/3 activity is essential for the activating cleavage of proglucagon, proopiomelanocortin, proinsulin, and proghrelin [45]. In our current study, *PCSK1* gene expression, which contributes to GLP-1 biosynthesis, is decreased in the jejunal epithelium of obese individuals with or without type2 diabetes, similar to as reported in L-cells of mice fed with HFD [41]. The expression of *PCSK2* was recently reported in the gut, where *PCSK1* and *PCSK2* gene expression was increased in each gut segments from obese-diabetic individuals compared to healthy individuals [46]. The expression of *PCSK2* in the human jejunum reported here is an argument in favor of the potential production, by the gut, of extrapancreatic glucagon reported in individuals with a total pancreatectomy [47]. GLP-1 secretion is triggered at the apical membrane by nutrients, such as lipids, via GPR119 and GPR40 [48]. *FFAR1* transcription is activated by PAX6 [49]. Here, *GPR119* and *FFAR1*, in EEC was lower in ObD individuals suggesting a reduced capacity to secrete GLP-1 in response to lipids.

Among cohorts in the current study, 23–52% of individuals were taking DPP4-inhibitors or GLP-1 receptor agonists. We cannot exclude that these antidiabetic therapies could influence EEC differentiation through intestinal hypertrophy as reviewed in [50]. However, here we observed an impaired EEC differentiation and GLP-1 cell density whose are in opposite with an intestinotrophic effect. Indeed, our study provides the novel finding that density of GLP-1-positive cells is reduced in jejunum in T2D individuals with severe obesity whereas CCK-, GIP- and PYY- producing cell densities remained unaffected. This could explain why after a challenge by meal tests, circulating GLP-1 concentration fails to increase appropriately in Ob and ObD individuals as previously reported [51, 52]. Although it has been shown a co-expression of GLP-1 and PYY in primary cultured human colon cells [53] and in human jejunum [54], the expression of a specific hormone in any particular EEC is dependent of its location along the cephalocaudal axis [22] and along the crypt-to-villus axis [55]. In our study, PYY cell density in the Ob group remained unchanged, in contrast to the density of GLP-1 cells suggesting that PYY and GLP-1 are not colocalized in the same EEC subtype in the jejunum. Thus, we cannot exclude that the absolute number of CCK-, PYY- and GIP-positive cells is enhanced in obesity.

Furthermore, it has been demonstrated that GLP-1 secretion rises instantaneously after surgery [56]. This is compatible with the decreased EEC differentiation in the current study if we hypothesize that the observed impairment of EEC lineage in diabetic individuals is only present in the jejunum and that after bariatric surgery foods arrive to more distal segments of the small intestine with intact or restored EEC differentiation.

Some limitations of our study should be noted. First, results are limited to jejunum and we cannot exclude an impact of T2D in other small intestine segments. Furthermore, antidiabetic treatments are given to ObD subjects without it being possible to determine their impact on gene expression. Second, we have to point on the sex unbalance of our two groups of Ob subjects. Individuals undergoing bariatric surgery have a gender difference (males 20% vs. females 80%) [57]. This disparity is lower in older obese individuals with more comorbidities [57]. Gender can influence the tissue expression of common genes [58, 59]. It has been shown that small intestine presents less differential gene expression due to gender than other tissues [58]. However, we cannot here exclude a sex-biased gene expression. Third, RNAseq analysis provides a transcriptomic signature of EEC in Ob according to their diabetic status but the diabetic status could in turn impairs transcriptomic profile of EEC. Thus we cannot conclude to a causal link between these two observations. Finally, our transcriptomic data were obtained with a novel cell sorting strategy to achieve EEC enrichment in humans that has not been described before.

In conclusion, our data reveal cell differentiation and transcriptional mechanisms by which individuals with obesity and T2D display reduced GLP-1 cell density and could have a reduced capacity to produce GLP-1 in response to a meal (Fig. 4). Therefore, it could be of interest to intervene on EEC lineage to circumvent the reduction of post-prandial GLP-1 production and improve diabetic outcomes.

## Supplementary information

Supplementary figures
