# Supplementary material for: Type 2 diabetes is associated with impaired jejunal enteroendocrine GLP-1 cell lineage in human obesity
Source: Int J Obes (Lond). 2020 Oct 9;45(1):170–83. doi: 10.1038/s41366-020-00694-1 (PMC7752761; doi:10.1038/s41366-020-00694-1)
Supplement: Supplementary file 1 — Supplementary figures [file 41366_2020_694_MOESM1_ESM.pdf]

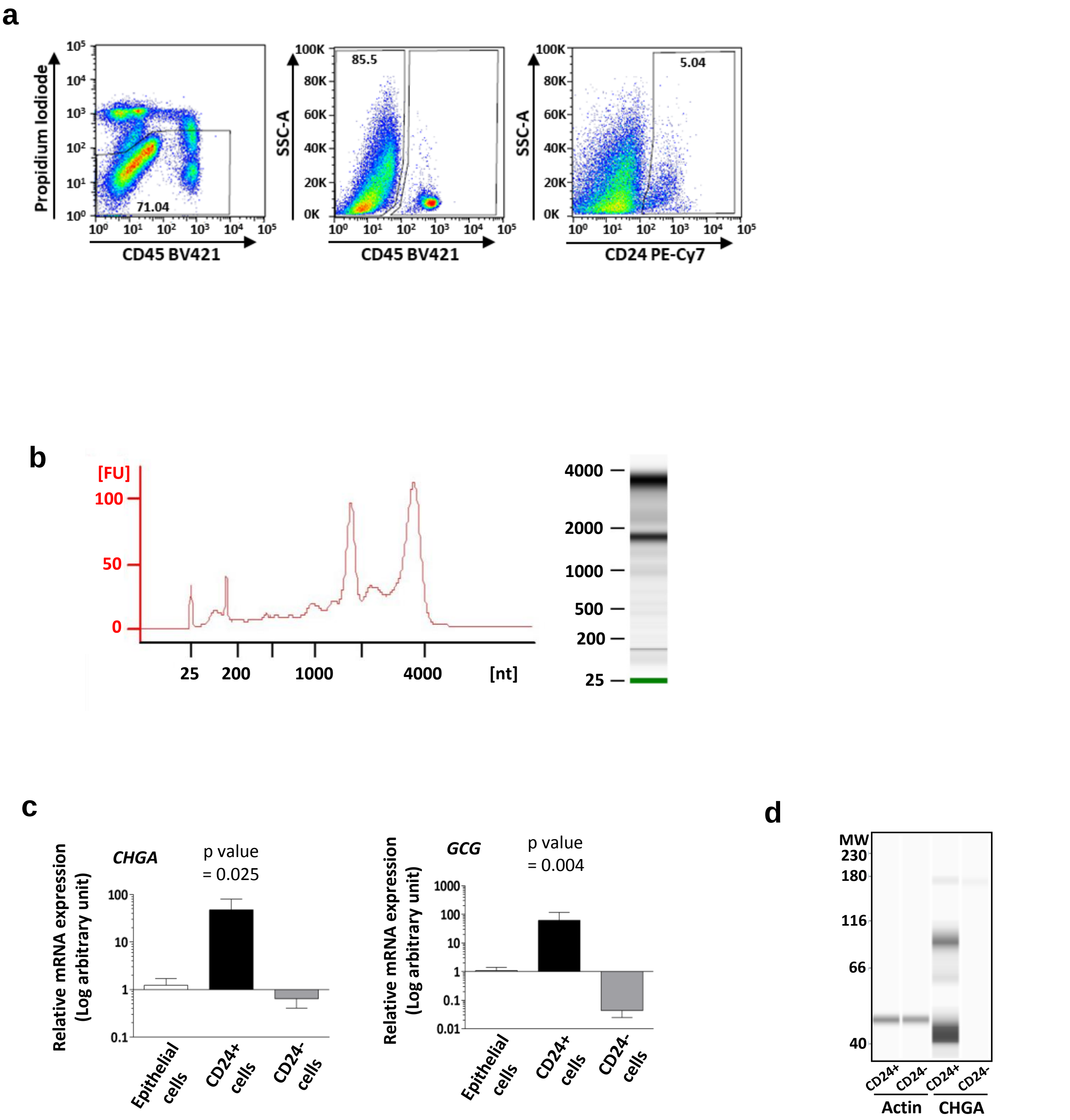

Supplementary Fig. 1 a) Gating strategy for EEC fraction isolation by FACS with CD45 and CD24 markers. **b)** Agilent Bioanalyzer 2100 trace of RNA quality from human EEC enriched populations. A representative RNA quality analysis of 8.2 value is presented (left panel). A representative electropherogram (lef panel) and the respective representation as migration profile (right panel). **c)** Relative mRNA expression of ECC markers Chromogranin A (CHGA, left) and Proglucagon (GCG, right) in total epithelial cells (open bar), CD24+ sorted cells (black bar) and CD24- sorted cells (gray bar). The mRNA levels were normalized by 18S mRNA levels. Results are expressed as mean ± SEM (n= 3 Ob individuals). **d)** Representative image by Simple Western assay of protein expression of CHGA (EEC marker) and Actin (control protein) in CD24+ and CD24- sorted cells (n= 4: 2 Ob and 2 ObD individuals).

Supplementary Figure 1
Osinski & al.

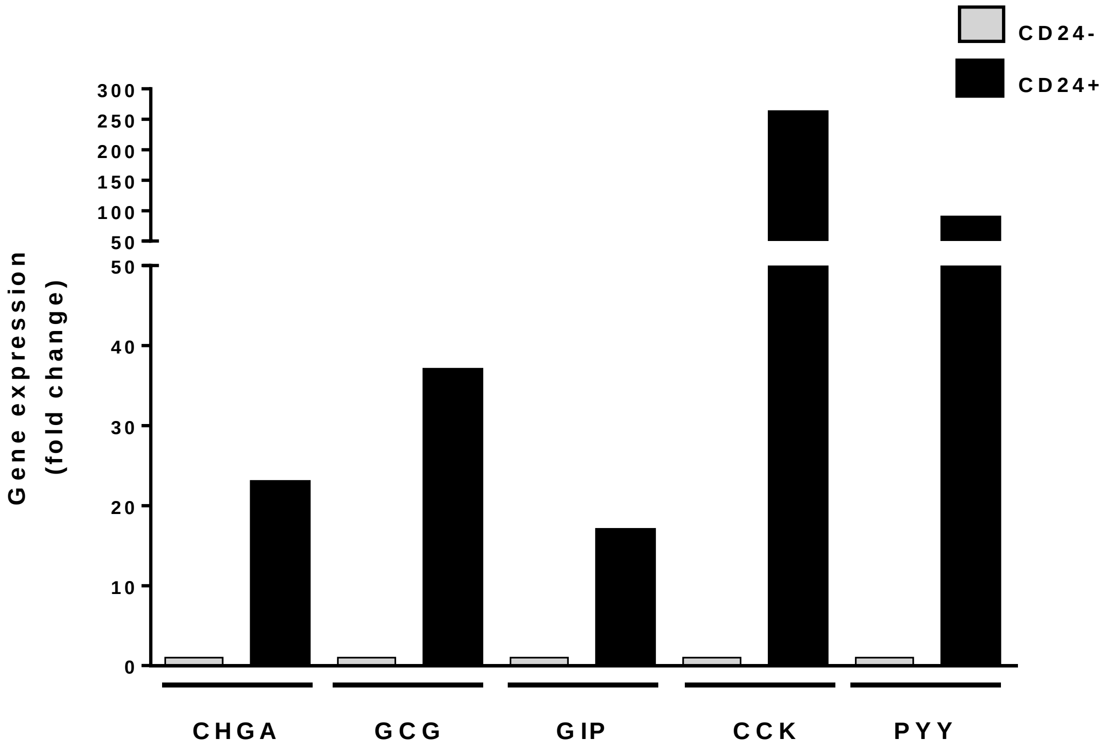

Supplementary Fig. 2 RNA sequencing analysis of enterohormone gene expression in CD24- (gray bar) and CD24+ (black bar) cells from one ObD subject. Values of gene expression in pseudo-counts are expressed as fold change.

Supplementary Figure 2
Osinski & al.

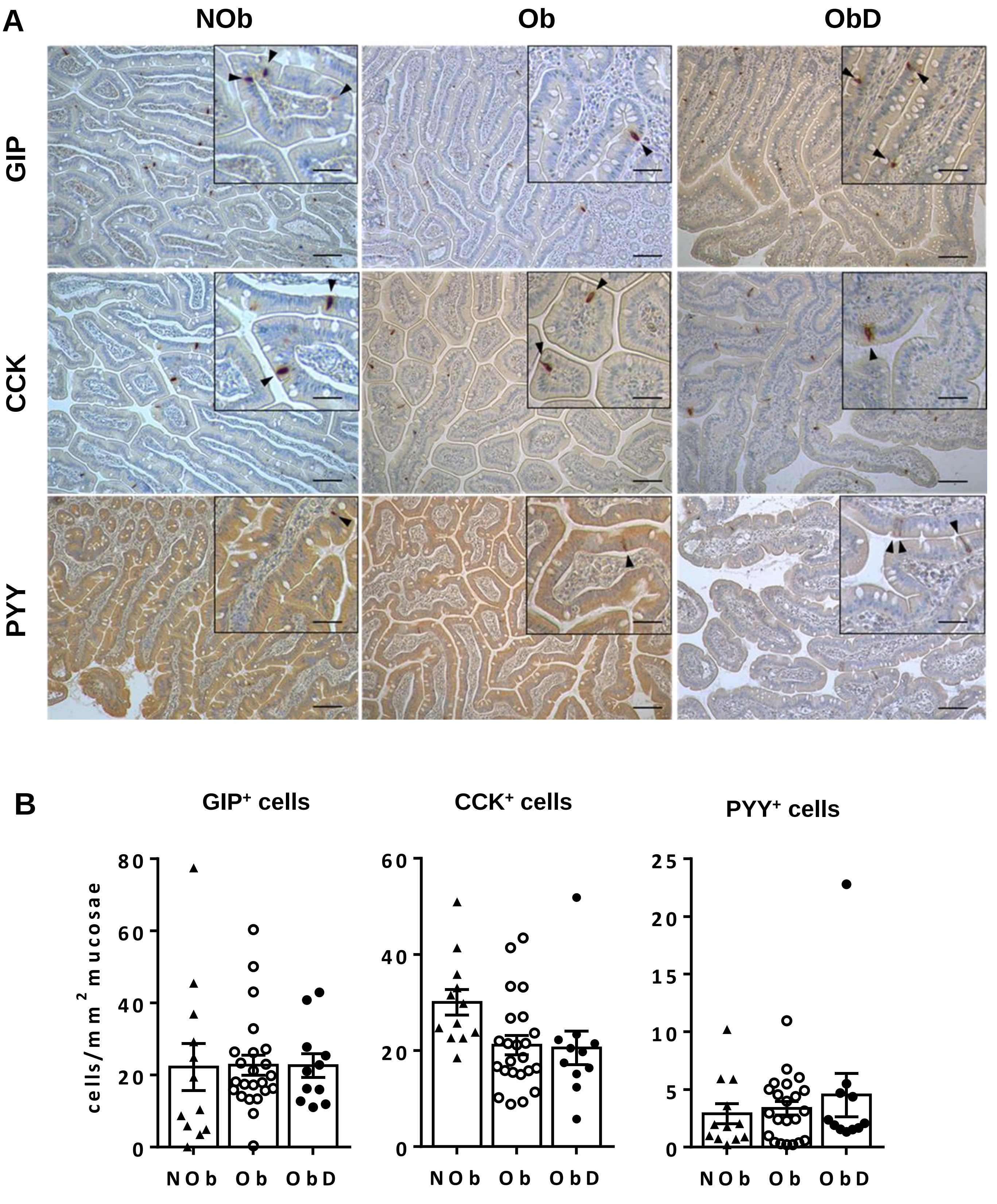

Supplementary Fig 3: EEC densities in non-obese (NOb), obese (Ob) and obese-diabetic (ObD) individuals. A) Representative images of GIP, CCK and PYY immunostaining in jejunum sections in which positive cells are colored in brown (scale bar 150 µm). A magnification is shown in the box (scale bar 300 µm) in which positive cells are indicated with black arrowheads. B) Cell densities are expressed as number of positive cells per mm^2 of the jejunum mucosae in NOb (n=12, black triangle), Ob (n=23, open circle) and ObD (n=11, black circle) individuals.

Supplementary Figure 3
Osinski & al.
